# Prostaglandin production selectively in brain endothelial cells is both necessary and sufficient for eliciting fever

**DOI:** 10.1073/pnas.2122562119

**Published:** 2022-10-17

**Authors:** Kiseko Shionoya, Anna Eskilsson, Anders Blomqvist

**Affiliations:** ^a^Division of Neurobiology, Department of Biomedical and Clinical Sciences, Linköping University, Linköping SE-58185, Sweden

**Keywords:** fever, cyclooxygenase-2, brain endothelial cells, microsomal prostaglandin E synthase-1

## Abstract

Fever is known to be elicited by prostaglandin E_2_ acting on the brain, but its origin has remained disputed. We show in mice that selective deletion of prostaglandin synthesis in brain endothelial cells, but not in neural cells or myeloid cells, abolished fever induced by intravenous administration of lipopolysaccharide and that selective rescue of prostaglandin synthesis in brain endothelial cells reinstated fever. These data demonstrate that prostaglandin production in brain endothelial cells is both necessary and sufficient for eliciting fever.

Pharmacologic inhibition or genetic deletion of the PGE_2_-synthesizing enzymes cyclooxygenase-2 (COX-2) and microsomal prostaglandin synthase-1 (mPGES-1) abolish immune-evoked fever (1, 2). Because both COX-2 and mPGES-1 have been shown to be induced in brain endothelial cells by systemic immune challenge (3), and because cell-specific deletion of these enzymes in the endothelial cells attenuates the febrile response (4), it has been hypothesized that fever is dependent on PGE_2_ produced by brain endothelial cells (5).

However, while the available data indicate that PGE_2_ produced in the brain endothelium participates in the febrile response to systemic immune challenge, they do not show whether such production is sufficient for the generation of fever or whether other sources are also required. Upon peripheral immune stimulation, PGE_2_ production is induced in several organs, such as liver and lung, and thus produced PGE_2_ has been suggested to be involved in, and even critical for, certain aspects of the febrile response (6). Furthermore, prostaglandin activation of peripheral nerves, including the vagus nerve, has also been implicated in the febrile response (7).

Here we examined whether fever could be elicited when induced prostaglandin production was restricted to brain endothelial cells. We first examined the febrile response to i.v. injection of lipopolysaccharide (LPS) in wild-type (WT) mice and in mice subjected to pharmacologic blockage or cell-specific deletions of COX-2, the latter generated by crossing mice with conditional deletion of COX-2 with mice expressing Cre recombinase under specific promotors, including the thyroxine transporter Slco1c1, resulting in recombination selectively in brain endothelial cells (8); Nestin, recombining in neural cells; and LysM, recombining in myeloid cells. While the response of these mouse models to intraperitoneal injection of LPS previously has been reported (4), we here employed an i.v. administration route via an indwelling jugular catheter. This procedure enables performing injection without handling the animals, hence avoiding the handling stress that otherwise interferes with and obscures the immune-induced body temperature changes (9). We chose to inject LPS, which elicits a complex inflammatory response, including the endogenous release of proinflammatory cytokines such as interleukin (IL)-1β, IL-6, and TNF. While IL-1β is implicated in many fevers (10), its i.v. administration was not feasible in the present experiments because of its very rapid elimination in plasma (11); in our hands such administration accordingly yielded no or little fever.

## Results and Discussion

**Fig. 1.**
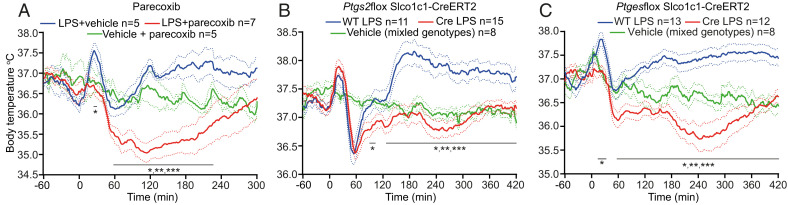
Body temperature responses following pharmacologic inhibition or cell-specific deletions of prostaglandin synthesis. (*A*) The selective COX-2 inhibitor parecoxib (10 mg/kg i.p.) was given 1 h before i.v. injection of LPS (30 μg/kg at time-point zero). *P < 0.05; **P < 0.01; **P < 0.001 for LPS + vehicle vs. LPS + parecoxib (two-way ANOVA for 0 to 300 min: *F*_2,13_ = 5.418, *P* < 0.05). (*B*) Selective deletion of the *Ptgs2* gene in brain endothelial cells. *P < 0.05; **P < 0.01; **P < 0.001 for WT LPS vs. Cre LPS (two-way ANOVA for 60 to 420 min: *F*_2,31_ = 12.36, *P* = 0.0001). (*C*) Selective deletion of the *Ptges* gene in brain endothelial cells. *P < 0.05; **P < 0.01; **P < 0.001 for Cre LPS vs. WT LPS (two-way ANOVA for 0 to 420 min: *F*_2,30_ = 20.52, *P* < 0.0001). Solid lines represent mean and dotted lines SEM.

As seen in Fig. 1, i.v. injection of LPS to WT mice (30 μg/kg) resulted in a three-phasic fever (best illustrated in Fig. 1*B*), with a first temperature peak at around 20 to 30 min after injection; followed by a hypothermic response, a rapid temperature recovery (second phase); and finally a third phase beginning at about 2 to 3 h after injection.

When WT mice were given a systemic injection of the selective COX-2 inhibitor parecoxib (Dynastat; Pfizer) prior to the LPS injection, all febrile phases were abolished (Fig. 1*A*). In mice with deletion of the Cox-2 gene (*Ptgs2*) specifically in brain endothelial cells, generated with a tamoxifen-inducible Cre recombinase system, both the second and third phases were abolished; however, the first phase of fever remained intact (Fig. 1*B*), whereas mice with deletion of the gene encoding mPGES-1 (*Ptges*), which converts the COX-2 product PGH_2_ into PGE_2,_ selectively in brain endothelial cells abolished all phases of fever (Fig. 1*C*). Mice with deletion of COX-2 specifically in neural cells or in myeloid cells showed the same temperature response to i.v. LPS as did WT mice.

**Fig. 2.**
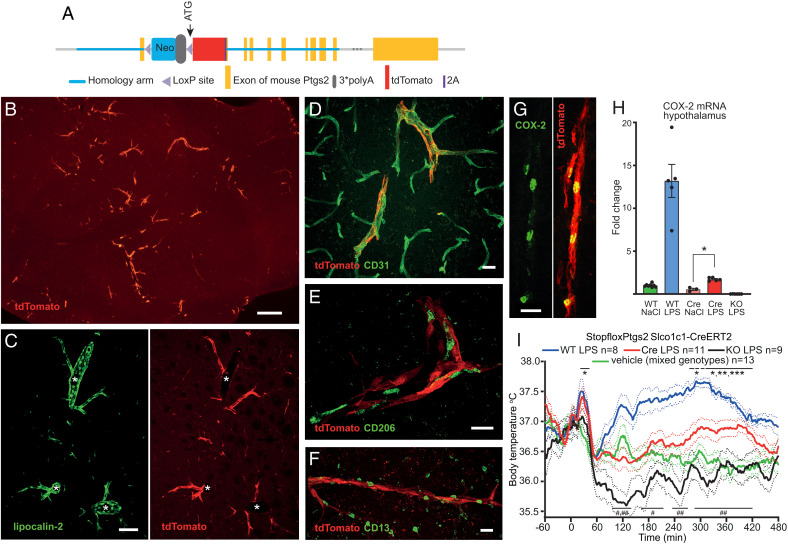
Fever occurs after selective expression of the *Ptgs2* gene in brain endothelial cells. (*A*) The targeted allele in Stopflox*Ptgs2* mice. (*B*) tdTomato immunofluorescent blood vessels in LPS-injected Stopflox*Ptgs2 Slco1c1*-CreERT2 mouse. (*C*) Dual labeling for lipocalin-2 (green) and tdTomato (red) in brain blood vessels. Lipocalin-2, but not tdTomato, is induced not only in small but also large vessel profiles (asterisks). (*D*) Dual labeling (yellow pseudocolor) of blood vessels for tdTomato (red) and the endothelial cell marker CD31 (green). (*E* and *F*) Blood vessels expressing tdTomato (red) and profiles immunoreactive for the perivascular macrophage marker CD206 (green; *E*), and the pericyte marker CD13 (green; *F*). The CD206 and CD13-ir profiles are distinct from the tdTomato-labeled vessel wall. (*G*) COX-2 immunoreactivity (green) in tdTomato (red)-expressing blood vessel. Yellow pseudocolor shows coexpression of the two fluorochromes. (Scale bars: *B*, 250 μm; *C*, 100 μm; and *D*–*G*, 20 μm.) (*H*) qPCR for COX-2 mRNA in the hypothalamus of WT mice, Stopflox*Ptgs2 Slco1c1*-CreERT2 mice (Cre), and Stopflox*Ptgs2*^+/+^ mice (KO) 3 h after i.v. injection of LPS (30 μg/kg body weight) or vehicle. *P = 0.04 (one-way ANOVA [Kruskal–Wallis] *P* < 0.0001). (*I*) Temperature recordings in Stopflox*Ptgs2 Slco1c1*-CreERT2 mice (Cre), Stopflox*Ptgs2*^+/+^ mice (KO), and WT mice, after i.v. injection of LPS (30 μg/kg body weight) or vehicle. *P < 0.05; **P < 0.01; ***P < 0.001 for Cre LPS vs. vehicle, and ^#^*P* < 0.05; ^##^*P* < 0.01 for Cre LPS vs. KO LPS (two-way ANOVA for 0 to 420 min: *F*_3,35_ = 15.06, *P* < 0.0001). Solid lines represent mean and dotted lines SEM.

We next constructed a mouse line with a *loxP*-flanked transcriptional blocker upstream of the *Ptgs2* ATG site (Fig. 2*A*)—resulting in disrupted COX-2 expression—and crossed mice from this line (Stopflox*Ptgs2*) with mice expressing tamoxifen-inducible Cre recombinase under the *Slco1c1* promoter (*Slco1c1*-CreERT2), which is active almost exclusively in brain endothelial cells (8) (for details, see *SI Appendix*). Using immunofluorescent detection of tdTomato, which is expressed in tandem with *Ptgs2* after rescue (Fig. 2*A*), we found strong LPS-induced expression selectively in small- to medium-sized vessels (10 to 20 μm in diameter) throughout the brain (Fig. 2*B*). Dual labeling for tdTomato and lipocalin-2, which is strongly induced in brain endothelial cells upon peripheral immune challenge (12), showed that the tdTomato-expressing vessels also expressed lipocalin-2. However, lipocalin-2 was also expressed in larger vessels not expressing tdTomato (Fig. 2*C*). Dual labeling for tdTomato and cell-specific markers confirmed that the *Slco1c1*-CreERT2–induced recombination occurred selectively in brain endothelial cells (Fig. 2 *D*–*F*).

Consistent with the above findings, immune-induced COX-2 immunoreactivity, which colocalized with tdTomato, was seen perinuclearly in endothelial-like cells in small- to medium-sized blood vessels of LPS-injected Stopflox*Ptgs2 Slco1c1*-CreERT2 mice (Fig. 2*G*). qPCR showed COX-2 induction in the hypothalamus of Stopflox*Ptgs2 Slco1c1*-CreERT2 mice, like what was found in WT mice. However, both the basal levels of COX-2 messenger RNA (mRNA), as well as the LPS-induced COX-2 levels, were lower in the Stopflox*Ptgs2 Slco1c1*-CreERT2 mice than in the WT mice (Fig. 2*H*), as would be expected since recombination did occur only in a subset of all immune-activated endothelial cells. Stopflox*Ptgs2* mice without Cre (KO) showed very low levels of COX-2 mRNA, as expected. No significant recombination was found in lung or liver from the Stopflox*Ptgs2 Slco1c1*-CreERT2 mice, with mRNA levels of COX-2 being about 1‰ of those in WT mice (lung) or undetectable (liver), and no induced levels of PGE_2_ metabolites were found in plasma, as measured 20 min after i.v. injection of LPS, in contrast to what was seen in WT mice (WT, 810 ± 148 [SEM] pg/mL; Stopflox*Ptgs2 Slco1c1*-CreERT2, 401 ± 58 pg/mL; KO, 410 ± 21 pg/mL; naive, 356 ± 58 pg/mL).

Intravenous injection of LPS in Stopflox*Ptgs2 Slco1c1*-CreERT2 mice (Fig. 2*I*) resulted in a first phase of fever, as in WT mice. While there was no elevation of the body temperature during the second phase, the body temperature did not drop in contrast to what was seen in KO mice but was maintained at the same level as in mice injected with saline. During the third phase there was an elevated body temperature in the LPS-injected Stopflox*Ptgs2 Slco1c1*-CreERT2 mice compared to what was seen in the saline-treated mice.

It has long been discussed where the PGE_2_ synthesis responsible for the generation of fever upon systemic immune challenge occurs, and while it is generally agreed that brain endothelial cells play an important role, other sources of PGE_2_, such as peripheral and central macrophages, as well as prostaglandin- or cytokine-induced neural signaling, have been suggested (5–7, 13). We here demonstrate that prostaglandin synthesis by COX-2 expressed by a subset of brain endothelial cells (i.e., endothelial cells in small- to medium-sized, but not large blood vessels) is both necessary and sufficient for the generation of fever upon peripheral immune challenge with LPS. When COX-2 was selectively deleted in brain endothelial cells, the febrile response except its first phase was abolished, and when COX-2 was selectively rescued in brain endothelial cells of mice otherwise lacking COX-2, fever appeared. Furthermore, the finding from mice with mPGES-1 deletion in endothelial cells shows that endothelial PGE_2_ production is necessary for all phases of fever, including the first phase, which previously has been suggested to depend on other mechanisms, such as the synthesis of blood-borne PGE_2_ by macrophages in, e.g., the lung (6). However, as shown here the first phase occurred also in the absence of COX-2–induced peripheral PGE_2_ synthesis. While it was dependent on COX-2, as shown by its disappearance when COX-2 was inhibited with parecoxib, and while the selective expression of COX-2 in brain endothelial cells was sufficient for LPS to evoke the first phase of fever, COX-2 in brain endothelial cells was not necessary for that response. Additional sources for the COX-2 product that subsequently is converted to PGE_2_ by mPGES-1 in the endothelial cells could be perivascular macrophages (14).

## Materials and Methods

Detailed descriptions are provided in *SI Appendix*. In brief, mice with cell-specific deletion/expression of prostaglandin-synthesizing enzymes were generated using a Cre recombinase system. The animals were immune challenged by injection of lipopolysaccharide via an indwelling jugular catheter and body temperature was recorded via telemetry. Immunohistochemistry was done on free-floating frozen sections from formaldehyde-fixed tissue using indirect fluorescence. Plasma prostaglandins were analyzed using enzyme immunoassays and quantitative PCR was performed with TaqMan assays.

## Supplementary Material

Supplementary File

## Data Availability

All study data are included in the article and/or *SI Appendix*.
