# Supplementary material for: Prostaglandin production selectively in brain endothelial cells is both necessary and sufficient for eliciting fever
Source: Proc Natl Acad Sci U S A. 2022 Oct 17;119(43):e2122562119. doi: 10.1073/pnas.2122562119 (PMC9618080; doi:10.1073/pnas.2122562119)
Supplement: Supplementary File [file pnas.2122562119.sapp.pdf]

Supporting information Text

Extended Methods

Animals. A mouse *Ptgs2* conditional knockin was created (Cyagen Biosciences), using C57BL/6 ES cells for gene targeting. In the targeting vector, a *LoxP*-Neo cassette with 3*polyA-*LoxP* was placed upstream of the mouse *Ptgs2* ATG start codon with 7-10bp. A tdTomato (without start and stop codon)-2A cassette was placed downstream of the mouse *Ptgs2* ATG start codon. Heterozygous targeted mice were intercrossed to create homozygous mice, which were bred with a line expressing tamoxifen inducible CreERT2 from the *Slco1c1* promoter (1) to generate mice with selective expression of COX-2 in brain endothelial cells.

Mice with conditional deletion of COX-2, possessing *loxP* sites flanking exons 4–5 of the *Ptgs2* gene (2), or of mPGES-1, possessing *loxP*-sites flanking exon 2 in the *Ptges* gene (3), were crossed with *Slco1c1*-CreERT2 to create mice with selective deletion of COX-2 and mPGES-1, respectively, in brain endothelial cells. The mice with conditional deletion of COX-2 were also crossed with Nestin-Cre or LysM-Cre lines (4, 5), to generate mice with selective deletion of COX-2 in neural or myeloid cells.

The mice were housed under constant ambient temperature (21°C), on a 12 h/12 h light/dark cycle (lights on at 7 A.M.) with food and water available *ad libitum*. Gene deletion in mice with the *Slco1c1* CreERT2 construct was induced by intraperitoneal injection of tamoxifen (1 mg tamoxifen in a mixture of 10% ethanol and 90% sunflower seed oil twice a day for 5 d) at least 5 weeks before additional experiments. The same treatment was given to offspring not expressing Cre.

Surgery. Mice were anesthetized with ketamine (70 mg/kg) and dexmedetomidine (0.4 mg/kg) and implanted i.p. with a transponder that records core body temperature (E-Mitter; Starr Life Sciences). During the same session, the mice were provided with an indwelling jugular catheter that was exteriorized at the back of the neck and connected to a swivel system (CMA Microdialysis) on the top of the cage, permitting injections without handling the mice (for details, see (6)). Following the surgery, the mice were kept at an ambient temperature of 29°C, providing near-thermoneutral conditions (7) and after 3 d injected with LPS (30 μg/kg), or saline, via the indwelling intravenous catheter.

All animal experiments were approved by the Animal Care and Use Committee at Linköping University (#1854/2018)

Immunohistochemistry. Following transcardial perfusion with a phosphate-buffered (0.1 M; pH 7.4) paraformaldehyde solution (4%), tissue of interest was postfixed for 3 h, cryoprotected with 30% sucrose in PBS, and cut at 30 μm on a freezing microtome. Primary antibodies were rabbit or goat anti-RFP (Abcam, 1:1,000; Origene, 1:500), detecting tdTomato; goat anti-CD13 (R&D Systems, 1:120), labeling pericytes (8); rat anti-CD206 (AbD Serotec, 1:500), labeling perivascular macrophages (9); rat anti-CD31 (AbD Serotec, 1:1000), labeling endothelial cells (10); goat anti-lipocalin-2 (R&D Systems, 1:500); and rabbit anti-COX-2 (Santa Cruz, 1:500). Secondary antibodies were Alexa 555 donkey anti-rabbit, Alexa 555 donkey anti-goat, Alexa 488 donkey-anti rat (Life Technologies, all diluted 1:1000), and, for COX-2 in Stopflox*Ptgs2 Slco1c1*-CreERT2 mice, donkey anti-rabbit HRP conjugate (Dako, 1:500) followed by tyramide signal amplification (PerkinElmer).

Microscopy. Fluorescent images were acquired with a Nikon Eclipse 80i microscope, equipped with epi-fluorescence and fluorescein and rhodamine filters (Nikon B-2A and G-2A), a Nikon DS-Ri1 digital camera, and Nis-Elements software, using 10x/0.45 and 20x/0.75 lenses. Confocal images were acquired with a Zeiss LSM700 confocal microscope with 488 and 555 nm lasers, 10x/0.45 and 40x/1.3 lenses, and Zen black 2.3 software. Image processing, restricted to adjustment of brightness and contrast, was performed in Adobe Photoshop.

Immunoassay. Mice were killed by asphyxiation with carbon dioxide, after which blood was drawn from the right atrium, transferred to EDTA-coated tubes (Sarstedt) to which was added indomethacin (10 μm; Sigma-Aldrich), and centrifuged at 7000 × *g* for 7 min at 4°C. The plasma was immediately frozen on dry ice and kept at −70°C. The concentration of PGE_2 metabolites in plasma (diluted 1:20) was determined with a Prostaglandin E Metabolite EIA Kit (Cayman Chemical). The values were calculated using a standard curve ranging from 0.2 to 50 pg/ml (R^2 = 0.999). The kit antiserum recognizes derivatized 13,14-dihydro-15-ketoPGE_1, 13,14-dihydro-15-ketoPGE_2, and bicycloPGE_1, but has <0.01% cross-reactivity with arachidonic acid, leukotriene B4, tetranor-PGEM, tetranor-PGFM, PGD_2, PGE_1, 6-keto PGE_1, PGE_2, $PGF_1α$, 6-keto $PGF_1α$, $PGF_2α$ and thromboxane B_2.

Real-time PCR. The mice were killed by asphyxiation with CO_2. Brain regions dissected were placed in RNAlater stabilization reagent (Qiagen) and kept at −70°C until further processing. RNA was extracted with RNeasy Plus Universal Kit (Qiagen), and reverse transcription was done with High-Capacity cDNA Reverse Transcription Kit (Applied Biosystems); qPCR was performed using Gene

Expression Master Mix (Applied Biosystems) on a 96-well plate (7500 Fast Real-Time PCR System Software; Applied Biosystems). Mm00478374_m1 (COX-2), and Mm99999915_g1 (GAPDH) TaqMan assays (Applied Biosystems) were used.

Statistics. In all comparisons, littermates were used. Groups were designed to be age and sex matched, but within these limitations, animals were randomly assigned to the respective group. Statistical analyses were done in Prism version 9 (GraphPad). Significant differences were determined using a one-way or two-way ANOVA, followed by a *post hoc* test with correction for multiple comparisons controlling the false discovery rate according to the linear step-up multiple-testing procedure (11). Results were considered statistically significant when $p < 0.05$.

SI References

1. D. A. Ridder *et al.*, TAK1 in brain endothelial cells mediates fever and lethargy. *J. Exp. Med.* **208**, 2615-2623 (2011).
2. T.-o. Ishikawa, H. R. Herschman, Conditional knockout mouse for tissue-specific disruption of the cyclooxygenase-2 (Cox-2) gene. *Genesis* **44**, 143-149 (2006).
3. D. B. Wilhelms *et al.*, Deletion of prostaglandin E2 synthesizing enzymes in brain endothelial cells attenuates inflammatory fever. *J. Neurosci.* **34**, 11684-11690 (2014).
4. F. Tronche *et al.*, Disruption of the glucocorticoid receptor gene in the nervous system results in reduced anxiety. *Nat. Genet.* **23**, 99-103 (1999).
5. B. E. Clausen, C. Burkhardt, W. Reith, R. Renkawitz, I. Förster, Conditional gene targeting in macrophages and granulocytes using LysMcre mice. *Transgenic Res.* **8**, 265-277 (1999).
6. L. Engström *et al.*, Lipopolysaccharide-induced fever depends on prostaglandin E2 production specifically in brain endothelial cells. *Endocrinology* **153**, 4849-4861 (2012).
7. A. Y. Rudaya, A. A. Steiner, J. R. Robbins, A. S. Dragic, A. A. Romanovsky, Thermoregulatory responses to lipopolysaccharide in the mouse: dependence on the dose and ambient temperature. *Am J Physiol Regul Integr Comp Physiol* **289**, R1244-1252 (2005).
8. F. Alliot, J. Rutin, P. J. Leenen, B. Pessac, Pericytes and periendothelial cells of brain parenchyma vessels co-express aminopeptidase N, aminopeptidase A, and nestin. *J Neurosci Res* **58**, 367-378 (1999).
9. I. Galea *et al.*, Mannose receptor expression specifically reveals perivascular macrophages in normal, injured, and diseased mouse brain. *Glia* **49**, 375-384 (2005).
10. E. Thorp, T. Iwawaki, M. Miura, I. Tabas, A reporter for tracking the UPR in vivo reveals patterns of temporal and cellular stress during atherosclerotic progression. *J Lipid Res* **52**, 1033-1038 (2011).
11. Y. Benjamini, A. M. Krieger, D. Yekutieli, Adaptive linear step-up procedures that control the false discovery rate. *Biometrika* **93**, 491-507 (2006).
